# Gut Health and Influencing Factors in Pigs

**DOI:** 10.3390/ani13081350

**Published:** 2023-04-14

**Authors:** Csaba Szabó, James Kachungwa Lugata, Arth David Sol Valmoria Ortega

**Affiliations:** 1 Department of Animal Nutrition and Physiology, Faculty of Agriculture and Food Sciences and Environmental Management, University of Debrecen, Böszörményi Street 138, 4032 Debrecen, Hungary; 2 Doctoral School of Animal Science, Faculty of Agriculture and Food Sciences and Environmental Management, University of Debrecen, Böszörményi Street 138, 4032 Debrecen, Hungary

**Keywords:** pig, gastrointestinal tract, feed additives

## Abstract

**Simple Summary:**

The gastrointestinal tract of any organism is not only important from a digestion and nutrient absorption point of view, but it interacts in many ways with metabolism. Our knowledge regarding these connections is increasing continuously. Animals capable of intensive production to supply sufficient food are the most vulnerable to disruptions in the gut. Therefore, the knowledge of effective feed additives which can be used to avoid or cure these problems and their consequences is important in modern animal production systems.

**Abstract:**

The gastrointestinal tract (GIT) is a complex, dynamic, and critical part of the body, which plays an important role in the digestion and absorption of ingested nutrients and excreting waste products of digestion. In addition, GIT also plays a vital role in preventing the entry of harmful substances and potential pathogens into the bloodstream. The gastrointestinal tract hosts a significant number of microbes, which throughout their metabolites, directly interact with the hosts. In modern intensive animal farming, many factors can disrupt GIT functions. As dietary nutrients and biologically active substances play important roles in maintaining homeostasis and eubiosis in the GIT, this review aims to summarize the current status of our knowledge on the most important areas.

## 1. Introduction

The pigs’ gastrointestinal tract (GIT) or gut is vital in processing food materials into absorbable nutrients that their body needs for maintenance and production. Aside from its digestive and absorptive function, the pigs’ GIT is vital in maintaining immune homeostasis. Their intestine is considered the largest immune organ in their body, accounting for more than 70% of the immune cells in the body [1,2,3]. Moreover, the porcine intestine has many microorganisms contributing to intestinal mucosal immunity. There is an interplay between the gut microbiome and the gut immune system, as the latter can regulate intestinal microorganisms’ distribution and composition and intestinal microbiota homeostasis by secreting various immune effector factors. Conversely, intestinal microorganisms can also promote the differentiation of immune cells, including regulatory T cells, through their specific components or metabolites [4]. Therefore, a fully functional and healthy GIT or gut is critical for the pigs’ welfare and production efficiency at every stage of life [5]. A healthy GIT can be achieved when the pig has effective digestion and absorption of food, the absence of gastrointestinal illness, normal and stable intestinal microbiota, an effective immune system, a state of well being, and is less susceptible to pathogenic bacteria and viruses [6,7]. Although the pigs’ GIT serves immune functions, it is still vulnerable to disruptions in any pig’s life. Persistent challenges are evident in modern pig production, which is relatively due to diet, management, and the living environment of the pigs [4]. Pigs at different stages of life can face gut-associated problems often exacerbated by the modern pig production system. Piglets that are nowadays weaned at an early age with immature intestines and not fully developed gut microbiota are vulnerable to oxidative stress and destruction of the epithelial barrier and villus structures in the jejunum upon exposure to dietary and environmental factors, jeopardizing intestinal integrity and piglet performance [8]. For adult pigs (fatteners and sows), several factors can influence their GIT integrity. These include the presence of GIT pathogens, which produce toxins that harm the GIT. Moreover, changes in the diet, abuse of antibiotic treatments, and environmental stressors such as heat stress and cold stress can cause perturbation of commensal gut microbiota resulting in gut dysbiosis or damage the small intestinal mucosa, which can affect the pigs’ GIT functions and thereby compromise the pigs’ gut health demanding an efficient intervention strategy [4,9,10,11].

Nutrition is integral to the pig’s life because it affects the animal’s overall performance and can provide solutions to gut-related problems. Nutritional intervention strategies have been applied to improve the gut health and productivity of pigs challenged by various stressors such as changes in the diet, bacterial infection, and environmental stressors. The goals of such strategies are to improve nutrient digestion and absorption, regulation of gut microbiota to more favorable bacterial species, and immune modulation to enhance disease resistance, all of which are attainable with the supplementation of amino acids, phytogenics (essential oils), prebiotics, probiotics, and organic acids [12,13,14,15]. With the constant challenges in pig production, particularly in the maintenance of gut health, it is, therefore, the focus of this paper to review the causes of gut disruptions and dysbiosis in piglets, fattening pigs, and sows, as well as the alleviation capabilities of various dietary supplements.

In order to collect the available information on gut health in pigs, we searched for studies in the following databases: ISI Web of Knowledge, PubMed, Google Scholar, and Science Direct. We used the following search terms: (pig or swine or piglet or sow) and (GIT or gut or mouth or stomach or intestine or caecum or colon). Various combinations of the search terms were made (no date or language restriction for the selection) to find as many articles as possible. The articles falling into the scope of the manuscript have been saved and then classified according to the section of the GIT it deals with. The members of the author team were responsible for a certain part of the GIT to review the manuscripts found and prepare the sections according to the pre-determined structures. After that, the corresponding author compiled the sections and formatted them according to the journal template.

## 2. Mouth and Stomach

### 2.1. Normal Functioning

The first part of the gastrointestinal tract is the mouth. Generally, in pigs, little attention is paid to its functions and importance since pigs are considered weakly chewing animals (with little impact on particle size reduction), and feed remains for a very short time in the mouth. Therefore, average feed particle size is important because it directly affects digestibility and digesta transit, gelation, and content segregation into liquid, solid, and fibrous parts in the stomach [16]. Saliva contains epithelial growth factors, mucins and prostaglandins that protect the esophagus [17,18,19]. The electrolyte and water content of saliva is also important from the thermoregulation and body homeostasis point of view. Any active compound found in the saliva can express its effect on the stomach. Even if it is known that saliva contains amylase, which is activated by Cl^−^, it was considered that carbohydrate digestion in the stomach is insignificant due to the low pH. However, recently, it has been demonstrated that gastric starch digestion plays an important role in pigs [20]. Pigs demonstrated the ability to model human meal starch digestion [21,22].

The low gastric pH and the acidification of feed consumed play an important role in the defense mechanisms against pathogenic microbes [23]. The corpus region of the stomach has a significantly higher expression of genes involved in acid and enzyme production, while the antrum has significantly higher gastrin expression [24].

### 2.2. Disruptions

One of the major problems with gastric function is the need for more acidity, which impairs protein digestion and the elimination of harmful microorganisms. This situation mainly happens around weaning, when the glandular part of the stomach is still not functioning fully [25]. Moreover, the higher buffering capacity of high protein feeds also decreases acidity. Therefore, an aim of modern piglet feed formulations is to decrease protein levels to about 18%. In older pigs (sows and finishers), gastric ulcers had a high incidence [26]. The syndrome can be caused by several factors (single or in combination): starvation, ad libitum feeding, too fine feed particles, feed nutrient content, highly fluidic gastric content, and accompanying gastric bacterial infections [25,26]. However, about one-third of the nursery pigs also presents ulcers [25].

### 2.3. Dietary Interventions

#### 2.3.1. Particle Size

Fractionated gastric content and its diverse pH values play an important role in preventing esophageal ulcers in pigs. Coarse feed increased mean retention time and improved the fractioning of gastric content [16]. Larger particle size resulted in higher pH at the proximal part of the stomach than at the distal part [21]. This also can be manifested in altered microbiota (members of the genera *Lactobacillus* and *Mitsuokella* and propionate and butyrate producers are stimulated) [27]. Not only can particle size have a beneficial effect, but also higher fiber content. Even straw bedding (consuming straw) can markedly reduce the incidence of gastric lesions [28]. However, the increased fiber intake may reduce carcass yield [29]. Elevated β-glucan levels in fee result in less fractioning of stomach content [30]. More solid gastric content significantly reduced the risk of gastric ulcers in nursery pigs; however, notable individual variation was also observed [25]. Similar results were obtained with heavy pigs fed whole-ear corn silage [31]. The main limitation of the studies reviewed is that the mean particle size was not instrumentally measured. Therefore, a precise conclusion on ulcer preventive particle size cannot be reached. In one study, the average particle size of 10 farms’ feed was around 554 µm, and the average prevalence of gastric ulcers was 35.5% [25]. This suggests that a larger particle size would be more beneficial, but the effect should be evaluated in context. For instance, the effect on pellet quality (durability), fattening performance, and slaughter quality (lean meat percentage) should be evaluated as well.

#### 2.3.2. Organic Acids

Organic acids are the most widely used feed additives, especially in young pigs with limited hydrochloric acid production. The primary effect of organic acids is to lower the pH of the stomach, which promotes an increase in pepsin activity. This improves the digestibility of proteins and amino acids [32]. Reduced pH will cause slower gastric emptying and fewer pathogenic microorganisms [33]. This effect also prevails in the initial section of the small intestine. This is because insufficiently acidic stomach contents favor harmful bacteria’s survival and penetration into the small intestine, which can cause diarrhea and even death. The causative agents of post-weaning edema disease are enterotoxic *E. coli* strains, the damage of which can be significantly reduced by organic acid feeding [34]. Formic, propionic, and fumaric acids proved the most effective; however, the experiments’ results are quite contradictory. Although piglets can achieve improvements of up to 10–20% in terms of weight gain and feed conversion, research has reported several cases of neutral or negative effects. There can be many reasons for this. The composition of the feed greatly affects the expected result. Feed mixtures with a high buffer capacity (for instance, high limestone or crude protein content) can significantly limit the pH-lowering effect of organic acids. Age is also very important; in the case of 21 days of weaning, a significant positive effect was achieved for 2–3 weeks after the weaning, after which the production of hydrochloric acid in the stomachs of piglets turned out to be sufficient [35].

As energy prices hike, acidic preservations, including mold inhibitors of grains, gain the advantage. The natural and additive acid content of these feeds could affect the acidity of the stomach. However, an organic acid mold inhibitor did not influence the expression of genes involved in gastric acid secretion, mucosal defense, and appetite in the stomach of grower pigs [36]. These acidic feeds can control the multiplication of pathogens by lowering the pH.

#### 2.3.3. Vitamin U (Cabagin)

Vitamin U (a vitamin-like substance) is S-methyl methionine sulphonium chloride, with a suggested therapeutic effect on ulceration [37,38]. Vitamin U is found mainly in the plants of the *Brassicaceae* family and can be extracted from cabbages (hence its alternative name, cabagin). Although the development of mucosal lesions in the stomach of fattening pigs could not be prevented by regular feeding of vitamin U in small doses [39], the aggravation of the pathologic process was markedly inhibited, and the number of deaths due to severe ulcerative lesions was considerably reduced. In light of the high prevalence of gastric ulcers, new research can be suggested with this compound.

## 3. Small Intestine

### 3.1. Healthy Gut

The major site of macro- and micronutrient digestion and absorption is the small intestine (SI). The first and shortest section of the small intestine is called the duodenum. It plays a crucial role in food digestion by receiving partially digested foods from pancreatic and stomach secretions containing digestive enzymes. The jejunum accounts for nearly 80% of the small intestine in pigs. It has circular folds and villi to increase surface area for absorbing small nutrient particles digested by enzymes in the duodenum. The ileum is responsible for absorbing vitamin B12, re-absorption of conjugated bile salts, and other nutrients that the jejunum did not absorb [8,40,41]. The main digestive enzymes are amylase (breaks down starches and carbohydrates into sugars), lipase (breaks down lipids, which are fats and oils, into glycerol and fatty acids), and protease (breaks down proteins into amino acids). Trypsin and chymotrypsin are the two most significant protein-digesting enzymes in an animal’s intestine. Trypsin breaks down proteins into peptides by recognizing peptide bonds formed by arginine and lysine carboxyl segments. Chymotrypsin primarily degrades proteins and polypeptides into small peptides and amino acids. Chymotrypsin mainly acts on the peptide bond formed by the aromatic amino acids’ carboxyl group, with little activity on the peptide bonds formed by leucine, glutamine, and methionine. The activities of these enzymes are essential indicators of the animals’ digestive capability of different nutrients [42,43,44,45], thereby indicating a healthy intestine.

The animals’ enteric nervous system (ENS) with Its principal components (myenteric (Auerbach) and submucosal (Meissner) plexuses regulates the major enteric process in the intestine, such as detecting nutrients, immune response, motility, microvascular circulation, intestinal barrier function, and epithelial secretions of fluids and bioactive peptides [46]. ENS also cooperates with intestinal microbiota to maintain a stable intestinal microenvironment [47]. The guts mucus layer serves as the first line of defense against the infiltration of pathogenic microorganisms into the circulation by forming a barrier between the intestinal epithelium and the luminal content to prevent pathogenic invasion; such barrier could also prevent the commensal microbiota interacting with the gut [48]. Nevertheless, the intestinal microbiota interacts with the ENS and could regulate intestinal function [49]. This is believed to be possible through microbiotas’ production of molecules (synthesis and secretion) [47]. Microbe-associated molecular patterns (MAMPs), such as LPS on the surface of Gram-negative bacteria, are detected by Toll-like receptor 4 (TLR4), which is expressed in myenteric neurons. The activation of such receptors promotes intracellular signaling pathways and the production of cytokines responsible for activating the innate immune system. Moreover, it was suggested that Toll-like receptors regulate neuronal survival and neurogenesis of the ENS; hence, such LPS-mediated activation of TLR4 could influence the enteric neurons’ survival, regulate gut motility, and promote the integrity of the ENS [47,50].

The intestinal epithelium (IE) is a single-cell layer constituting the most significant barrier against the external environment. It is a selectively permeable barrier that permits the absorption of nutrients, electrolytes, and water while maintaining a strong barrier against intraluminal toxins, antigens, and enteric flora, preventing these substances from crossing the intestinal epithelium and entering the body [51]. The IEs’ functionality is mediated by the intestinal epithelial cells (IECs), which line the surface of the IE. The IEs’ composition (IECs: enterocytes, goblet cells, and Paneth cells) and their importance in nutrient absorption, immunoglobulin secretion, and synthesis and secretion of antimicrobial peptides and proteins and in maintaining intestinal homeostasis (SI microbiota eubiosis) through complex interactions, by which under such circumstances influence intestinal health, are well reviewed by Allaire et al. [52]. Literature in the study of gut health also emphasized the significance of microbiota composition; though less abundant in the SI than in the large intestine (LI), eubiosis in the SI can impact the gut health and the host animal [41,53,54,55].

The regulation of the intestinal epithelium components, enteric immune system, and eubiosis is essential in maintaining intestinal health. However, in young pigs, unlike adults (fatteners), the composition of gut microbiota and the regulation of these components are still premature, need careful attention, and are crucial for their survival. The mucosal immune system is responsible for preventing inappropriate immune reactions to food antigens or the commensal flora (beneficial microorganisms) and is the first immune defense barrier against pathogenic entry. At birth, the piglet is immunodeficient and highly dependent upon the supply of antibodies and immune cells (mainly macrophage and T cells) in maternal colostrum. Considering the rapid change from non-selective to selective uptake of macromolecules of the intestinal lumen, colostrum must be ingested within the first 24 h of the piglet’s life. This so-called passive immunity can provide short-term protection for piglets (21–30 days) depending on the level of sow antibodies absorbed by the piglet, and it was also found that the synthesis of antibodies by the piglet is influenced by the number of maternal antibodies that they absorbed [56,57]. Moreover, it has been reported that antibodies in colostrum could have a beneficial effect on the development of the gastrointestinal tract of neonatal piglets by improving their intestinal morphology [58]. Nevertheless, the piglet’s mucosal immune system develops over the first few weeks of life and is associated with intestinal microbiota colonization that drives immune maturation influencing the occurrence of effector lymphocytes (plasma cells and helper and cytotoxic T cells) and inhibiting colonization and overgrowth of the pathogen through the production of inhibitory substances such as bacteriocin. It is considered mature when piglets reach 6 weeks old as a large number of immune cells are prominent in the gut lymphoid tissue, but in the current intensive production system, piglets are weaned at a very young age (between 14 and 30 days) and are presented with a vast and diverse range of microbial and dietary/environmental antigens, which could be detrimental on gut immune homeostasis and can lead to the impairment of gut integrity [59,60].

### 3.2. Small Intestine Disruptions

Scientific works of literature regarding gut health have analyzed GIT characteristics (intestinal histology, morphology (influenced by prenatal and postnatal environment), barrier integrity (measurement of the transepithelial electrical resistance: TEER), immune response, and alteration of microbiome composition) in assessing gut health as these characteristics affect the functionality of the GIT, thereby affecting the performance of the animal [61,62,63,64,65]. Furthermore, the intestine’s certainty of exposure to foreign substances and microbial pathogens is a critical source of reactive oxygen species (ROS). Excessive generation of ROS can cause an imbalance in the animals’ antioxidant systems, leading to intestinal oxidative stress (OS). Such a stressor influences the early stage of intestinal injury and acts as an activating factor for intestinal barrier dysfunction, causing immune imbalance and inflammation [66,67,68]. Since intestinal OS causes intestinal inflammation, the levels of pro-inflammatory cytokines (including tumor necrosis factor-α [TNF-α], interleukin-1β [IL-1β], IL-6, IL-8, and interferon-γ) that are released from intestinal immune cells and mucosal epithelial cells upon increased production of ROS which functions as immunoregulatory molecules to activate immune cells causing inflammation are good biological indicators in assessing the intestinal condition. These pro-inflammatory cytokines redirect nutrients from growth to the immune system, which also compromises animal performance. Moreover, products of oxidative damage of lipids and proteins such as malondialdehyde (MDA) and carbonyls are known biological markers for OS [8,69,70]. Various stressors can be experienced by pigs in different stages of their productive life, which can affect their intestinal health, subsequently affecting their growth, performance, and productivity.

#### 3.2.1. Intestinal Development of Intrauterine Growth Restricted Pigs

Studies have reported that the uterine environment influences the intestinal development of animals. Hence, stressors experienced by the dam during pregnancy could compromise the functionality and integrity of their offspring’s intestinal health, which might have long-term adverse effects throughout the animals’ postnatal life [71]. Several stressors that sow face during pregnancy, such as heat stress (caused in utero heat stress (IUHS) and maternal malnutrition (occurs if the sows’ nutritional needs are not met, crucial during the last trimester of gestation as the nutritional needs of the sow to support fetal growth increases at this stage) can result in intrauterine growth restriction (IUGR), which is defined as impaired growth and development of the mammalian embryo or fetus or its organs during pregnancy [72,73,74,75]. There is an increasing number of evidence regarding the adverse effects of IUGR in the intestinal development of pigs. In the study of Ferenc et al. [76], IUGR piglets have lighter small intestines, shorter villi height, thinner muscularis of the jejunum, and higher expression of apoptosis markers in the intestine than normal body weight piglets. In another study, the adverse effects of IUGR on the intestinal development of pigs was observed during their growing period (70 days old), as pigs under such condition have short villus height to crypt depth ratio and low absorptive area, increased apoptosis, and low proliferation of duodenum epithelium. Moreover, IUGR pigs have low activity of chymotrypsin and amylase throughout their growth to the finishing period, which also affected their growth performance [77]. Additionally, IUGR could alter small intestinal microbial communities (*Firmicutes*, *Proteobacteria*, *Ruminococcaceae*, *Lactobacillus*, and *Ochrobactrum*) in growing to finish pigs [78]. The said information suggests that IUGR pigs had slower gut mucosa maturation and reduced utilization of nutrients, as supported by compromised intestinal morphology.

#### 3.2.2. Piglets: Weaning Stress

Weaning and the period that follows (post-weaning) are among the most stressful events in a pig’s life. They can cause intestinal and immune system dysfunctions, resulting in decreased pig health, growth, and feed intake, especially during the first week after weaning [79,80]. In modern pig production, piglets are weaned early, between 14 and 30 days, by which time their intestinal immune system is still premature. This makes them highly vulnerable to changes in their intestinal morphology, inflammation, and intestinal epithelial permeability. In addition, changes also occur in the activity of digestive enzymes, which can exacerbate the situation. These biological changes at weaning and post-weaning could have short- and long-term effects on pig health and growth [45,62,79,81]. Several studies have shown that weaning causes disruption of the piglets’ intestinal morphology and histology as characterized by a reduction of small intestine length and villus height [59,82]. Since villi increase the internal surface area of the intestinal walls to absorb nutrients, this function could be jeopardized upon weaning and post-weaning [83]. In such conditions, the passage of harmful substances such as bacteria from the lumen into the bloodstream is evident due to the loosening of tight junction (TJ) proteins (Zonula occludens-1 (ZO-1), occludin) which are considered permeability barriers [84,85]. Exacerbating such a condition is the occurrence of intestinal OS, which is associated with weaning. OS can impair the intestinal epithelium, microbiota and barrier function. Excessive ROS production affects the growth cycle of intestinal epithelial cells and could modify certain cellular proteins and activate the upregulation of pro-inflammatory cytokines, which may further deplete TJ protein expression and increase gut permeability [86,87,88].

In the study of Hu et al. [89], piglets weaned at 21 days showed a reduction in mRNA expression of TJ proteins, a decline in intestinal TEER, and upregulation of pro-inflammatory cytokines (TNF-α and IL-6). In another study, where OS markers were observed after piglets were weaned at 21 days old, they exhibited excessive generation of ROS ((nitric oxide (NO), hydrogen peroxide (H_2_O_2_)) and decreased concentration of antioxidant enzymes (proteins that catalyze the conversion of reactive oxygen species and their byproducts into stable, nontoxic molecules are the most critical defense mechanism against oxidative stress-induced cell damage) such as superoxide dismutase (SOD) and glutathione peroxidase (GSH-Px). Subsequently, increased MDA levels were observed in the weaned piglets [90,91]. Such an increase in permeability could allow the translocation of pathogenic bacteria and toxins into the blood circulation via paracellular transport due to disruptions of TJs and can lead to endotoxemia. In addition, weaning can cause dysbiosis in the SI of piglets as it can reduce the abundance of *Lactobacillus* spp. (key players in disease prevention), which can give way to an increase in the number of pathogenic bacteria such as *Salmonella* and Enterotoxigenic *Escherichia coli* (*ETEC*) and dramatically increase the risk of gastrointestinal diseases [41,92,93].

#### 3.2.3. Mycotoxins

Fumonisins produced by Fusarium, particularly fumonisin FB1 (FB1), and trichothecenes, particularly deoxynivalenol (DON), are the most well-known mycotoxins in terms of intestinal pig health. Fusarium verticillioides and *F. proliferatum*, common pathogens of maize, produce FB1. Ingestion of FB1 by piglets induces villus fusion and atrophy, affecting intestinal absorption of nutrients; it also causes alteration of cytokine profile and reduction of antibody response. DON produced by *F. graminearum* and *F. culmorum*, mainly in wheat, barley, and maize, causes toxic and immunotoxic effects in various cell systems. Its main effect at the cellular level is the inhibition of protein synthesis via DON binding to ribosomes. Similar to FB1, exposure to DON can up-regulate the expression of pro-inflammatory cytokines and inflammatory genes. At the intestinal level, DON intoxication can induce changes in claudins (TJ proteins) expression, promoting intestinal permeability [94,95]. Young pigs are highly affected by the adverse effects of FB1 and DON. In piglets, chronic ingestion of these mycotoxins, even in low doses, either individually or in combination through the contaminated feed, is detrimental to the animals’ health. FB1 and DON can induce intestinal morphological and histological changes as evidenced by villi atrophy and fusion, reduced villi height and cell proliferation in the jejunum, and a decrease in goblet cells and lymphocytes. These mycotoxins also significantly up-regulated the expression of pro-inflammatory cytokines (TNF-α) in the jejunum and ileum and subsequently reduced the expression of TJ proteins (occludin) in the intestine. This alteration in the intestine increases the translocation of pathogenic bacteria and may predispose piglets to infections by enteric pathogens [96,97].

#### 3.2.4. Environmental Stressors in Pigs

Digestive challenges are also evident in adult pigs. Despite having a mature gut, they are still vulnerable to digestive disorders, although much lesser than piglets. Environmental factors such as temperature, management, and pathogens could influence the intestinal health of pigs. Unfavorable environmental conditions such as high ambient temperature (HAT), which exceeds the thermal comfort of fattening pigs (between 15 and 25 °C), compromise their ability to control their internal temperature, leading to heat stress (HS). Pigs, however, are homeothermic animals and are capable of thermoregulation. Nevertheless, physiological processes involved in thermoregulation, an increase in vasoconstriction in their GIT to redistribute blood to the periphery to dissipate heat, can lead to a decrease of blood flow into the GIT and a decrease in the supply of oxygen, which leads to hypoxia and ultimately inflammation and OS. HS-induced intestinal hypoxia, inflammation (increase in pro-inflammatory cytokine), and OS (decrease in SOD, increase in MDA) can negatively affect the intestinal integrity and functionality of pigs. Pigs exposed to HS reduced their expression of TJ proteins, decreased TEER, and compromised intestinal morphology (reduced villi height and crypt depth), indicating intestinal permeability and promoting dysbiosis and endotoxemia [98,99,100,101].

#### 3.2.5. Pathogenic Stressors in Pigs

Exposure of pigs to pathogens is a major concern in the current intensive pig production system. Enteric diseases are the consequence of pathogen infection—pathogenic bacteria such as *Lawsonia intracellularis* are one of the most economically essential bacteria affecting growing pigs. *Lawsonia intracellularis* causes porcine proliferative enteropathy (Ileitis) as it infects the small intestine and the large intestine; it causes inflammation and thickening of the ileum and proximal colon, reducing nutrient absorption [102]. Intestinal permeability influenced by stressors can lead to the entry of pathogenic bacteria, causing intestinal lesions and compromised functionality, which degrade the animals’ performance, demanding an efficient and sustainable remedy [70].

### 3.3. Nutritional Strategies to Improve Pig Intestinal Health

#### 3.3.1. Dietary Protein, Amino Acid Levels, and Supplementation

Rodrigues et al. [70] stressed that high dietary protein (HDP) level in the diet could have a detrimental impact on the pigs’ gut health with presumptions of the undigested protein entering the large intestine, available for microbial fermentation and the possible influence of HDP level to support the proliferation of pathogenic bacteria (ETEC), by increasing the pH of the gut through the high buffering capacity of protein. Zhang et al. [103] reported that the HDP levels in piglets’ diet caused detrimental changes in their intestinal morphology, permeability, pro-inflammatory cytokine concentrations, and alteration in the microbial community and increased the incidence of post-weaning diarrhea. Several studies have pointed out that pigs fed low dietary protein reduced intestinal mucosa inflammation and pH. Low protein diets have led to a lower amount of protein reaching the lower GIT, inhibiting the proliferation of pathogenic bacteria, and reducing the incidence of post-weaning diarrhea under stress conditions such as weaning stress and pathogenic challenges [104,105]. However, the such reduction could also reduce the productive performance of piglets [106] with a detrimental impact on their ileal microbiota and impaired intestinal morphology (reduction of dietary protein from 18.83 to 13.05% in the diet) [107]. Aside from supplementation of essential amino acids (crystalline lysine, threonine, tryptophan, methionine, and valine) to counter the negative impact of low protein diets on growth [108], succeeding protein re-alimentation (an application based on the concept of compensatory growth: a phenomenon where animals exhibit faster growth rate after their nutritional levels return to normal due to prior malnutrition or artificial restriction) have shown positive results [109,110]. Shi et al. [45] reported that the adverse effects on intestinal health due to the 14-day low protein diet (13.05%) were compensated by returning the normal protein diet (18.83%) on pigs for a duration of 14 days of feeding. Pigs with compensatory growth exhibited an increased abundance of *Lactobacillus* and a reduced abundance of *Salmonella*, *Halomanas*, and *Pseudomonas* in the ileum; subsequently, improved ileal morphology and barrier functions were observed. Moreover, dietary peptides and amino acid supplementation can positively affect physiological parameters such as maltase activity, production of inflammatory cytokine and tight junction proteins, etc. (Table 1).

#### 3.3.2. Vitamins

The modern pig and its intensive production are highly exposed to challenges that lead to disrupted redox balance and uncontrolled inflammation. As previously mentioned, factors such as weaning and environmental conditions can lead to stress on the pig, affecting its intestinal health and performance. Supplementation of vitamins (A, B group, C, D, and E) and micro-minerals (selenium (Se) and zinc (Zn)) in the pigs’ diet has been found to have a positive impact on their intestinal health under challenge by the mentioned stressors. Vitamins A and D share several mechanisms for regulating intestinal immune functions, one of which is the fact that epithelial and immune cells express the vitamin A receptor (retinoic acid receptor, RAR) and the vitamin D receptor (VDR) [114,115]. Although vitamin A is known for its key role in vision, fetal development, and reproduction, it also significantly influences epithelial cells. The said influence can be attributed to vitamin A’s involvement in synthesizing glycoproteins, which are essential in the normal formation, development, and maintenance of epithelial cells [116,117]. Several studies have shown that vitamin A regulates intestinal barrier function and contributes significantly to the production of the mucus layer that lines the intestine. This was supported by its metabolites’ (retinoic acid) ability to induce the expression of TJ proteins (ZO-1, occludin, claudin-6, and claudin-7). Moreover, it is essential for developing adaptive immunity to intestinal microorganisms and is an anti-inflammation vitamin because it is critical in enhancing immune function. Hence, its deficiency can be detrimental to the animals’ intestinal health [118,119,120]. Vitamin A supplementation in piglets (14 days post-weaning) improved their intestinal morphology and activity of digestive enzymes, positively affecting the piglets’ intestinal function [88]. Although known for bone mineralization and calcium homeostasis, recent research has demonstrated that vitamin D plays an important role in maintaining a healthy epithelial barrier and gut microbiota through the reduction of pathogenic bacteria [114]. It also stimulates the expression of antimicrobial peptides and increases the production of anti-inflammatory cytokines, subsequently reducing the pro-inflammatory cytokines [121]. Dowley et al. [122] reported that weanlings supplemented with mushroom powder containing vitamin D_2_ (100 µg/kg feed) improved their intestinal morphology, increased the expression of anti-inflammatory cytokines and nutrient transporters, and decreased the expression of pro-inflammatory cytokines indicating intestinal health improvement after weaning. B vitamins are also important supplements for intestinal health. B vitamins are essential in the diversity and abundance of the gut microbiota. Although it can be synthesized by microbiota of the distal gut (colon), this is only in limited amounts and are not enough to supply the daily requirement of the host and the microbiota [123]; thus, supplementation could address such an issue. Pigs can synthesize vitamin C; however, their supplementation could benefit newly weaned piglets under stress. Because vitamin C is the most important water-soluble antioxidant in extracellular fluids, it can neutralize ROS in the aqueous phase before lipid peroxidation begins. Moreover, vitamin C supplementation has been proven to improve the immune responses of piglets after weaning [119,124,125]. Vitamin E, a fat-soluble dietary antioxidant, acts in its antioxidative function in intracellular membranes. Its capability to protect the animal from intestinal injury has been observed by Xu et al. [126]. They observed that in hypoxia-challenged rats, vitamin E supplementation significantly alleviated the stressors’ damage to the intestine and increased the serum concentration of endogenous antioxidant enzyme (SOD), subsequently decreasing the MDA and pro-inflammatory cytokines. Moreover, the supplementation also increased the IgA in the ileum and improved the expression of TJ proteins. In piglets, vitamin C supplementation has potent antioxidant properties upon immediate post-weaning stress. Vitamin E supplementation improves the antioxidant system of piglets during post-weaning, and their combination in the diet could have a beneficial effect on the piglets’ health challenged by weaning stress [127].

#### 3.3.3. Micro-Minerals

Micro-minerals such as Se and Zn have distinct antioxidant functions, which can help improve the intestinal health and condition of the animal. Se, known to enhance the antioxidant capacity and immunity of animals, can be differentiated into either inorganic or organic sources, the latter being more bioavailable (readily absorbed in the GIT) and having a higher threshold for toxicity than inorganic sources [128,129]. In the study of Liu et al. [44], supplementation of Se-enriched yeast (0.25 ppm Se) enhanced the piglets’ antioxidant capacity and immune function, suppressing their inflammatory response. In finishers, supplementation of selenomethionine (SeMet) with (0.25 ppm Se) enhanced the immunoglobulin serum concentration of pigs and influenced enhanced selenoprotein (antioxidant enzyme) gene expression [130]. Zn plays a vital role in several biological processes; it participates in intracellular signal transduction and cell proliferation, influencing cellular function, acid–base balance, oxidative resistance, and immune capacity. It also has been reported that dietary supplementation of organic Zn can reduce intestinal permeability and prevent loss of intestinal integrity under-challenged by weaning and HS. In weanlings, supplementation of zinc lactate (100 mg/kg in the diet) improved the intestinal morphology of weaned piglets (28 days post-weaning) with significantly higher villus height and villus height to crypt depth ratio (V/C) in the jejunum and higher concentration of goblet cells in the ileum than piglets without zinc lactate supplementation. The said supplementation also enhanced the expression of occludin and mucin 2 (MUC2), indicating intestinal barrier integrity [131]. In HS-challenged growing pigs, supplementation of Zn amino acid complex (ZnAA, 200 mg) alleviated the ill effects of HS on their intestinal integrity [132]. Moreover, regulated ZnAA (60 mg/kg) with the addition of zinc sulfate (60 mg/kg) supplementation can improve the intestinal barrier integrity and could prevent endotoxemia, as evidenced by the pigs’ higher ileum TEER than pigs without ZnAA supplementation [133].

#### 3.3.4. Other Feed Additives

The definition and beneficial effects of various other feed additives (enzymes, pre and probiotics, organic acids, and secondary plant metabolites (essential oils)) on the gut health and development of pigs were discussed in detail elsewhere [70,134,135]. Therefore, only representative examples of research findings are presented in Table 2. It can be pointed out that the use of these feed additives could not only improve nutrient utilization but also greatly influence the alleviation of intestinal dysfunction in pigs under certain stressful conditions. This is due to these additives’ bactericidal properties against pathogens and their influence in changing the expression of certain genes associated with TJ influencing certain signaling pathways of certain gastric cells and enzymes, subsequently promoting gut health.

## 4. Large Intestine

### 4.1. Healthy Gut

The large intestine is a long, continuous tube in the digestive system. A pig’s large intestines comprise the caecum, colon, and rectum. The large intestine of pigs performs physiological tasks such as absorbing fluids and electrolytes (water, vitamins, and minerals) and acting as a physical barrier against microbial invasion [149]. The health of the large intestine is important for overall digestive health, as it supports the absorption of nutrients and helps to eliminate toxins and waste from the body. A healthy large intestine is indicated by beneficial microflora/microorganisms. A healthy large intestine depends on a balanced microbiota or a healthy micro-ecosystem [150]. Microorganisms ferment endogenous secretions and undigested meal components, primarily dietary fiber (DF), lipids, and insoluble protein, in the large intestine. Only short-chain fatty acid (SCFA) and a few vitamins may be absorbed in this section, which helps the pigs supply nutrients [151].

### 4.2. Types of Threats/Causes of Large Intestine Disruptions

Large intestine disruption refers to a medical condition when the large intestine is blocked, torn, or has another irregularity. This condition can lead to digestion issues, pain, and discomfort in pigs. In addition, large intestine disruptions in microbiota balance are influenced by internal (host) factors and external factors such as environmental, dietary, and social factors. The imbalance between the intestinal microbiota is also called dysbiosis [93]. There are several causes of large intestine disruptions in pigs, categorized into two main types: infection causes and non-infection (toxic causes and nutritional causes/deficiencies).

Infectious causes of pig large intestine disruptions include parasites, viruses, and bacteria. Parasites, such as the tapeworm, can cause the pig to become malnourished and lead to blockages in the large intestine due to the worms’ eggs being laid in the intestine. Viruses, such as porcine circovirus type 2 (PCV2), can cause inflammation and lesions in the large intestine, leading to disruption. Bacterial infections, such as salmonellosis, can lead to large intestine disruption due to the inflammation caused by the infection [152]. In addition to Salmonellosis infection caused by *Salmonella enterica serovar Typhimuirum*, *Escherichia coli* (especially enterotoxigenic *E. coli*-ETEC) is the most known cause of postweaning diarrhea in piglets, responsible for 50% of death per year globally [93].

Non-infectious causes of pig large intestine disruptions include dietary, immunological, and physiological factors. Dietary factors, such as the inclusion of high-fiber diets or diets containing too much fat, can disrupt the large intestine due to difficulty digesting the diet components [153]. Immunological factors, such as an imbalance of gut microflora or an overactive immune response, can lead to disruption due to the inflammatory nature of the imbalance. Lastly, physiological factors, such as genetic predisposition, can disrupt the large intestine due to the pig’s inability to digest or absorb certain nutrients [154].

Large intestinal inflammation (colitis) is the large intestine disruption that causes diarrhea in growing pigs and is believed to be caused by infection/pathogens and dietary factors [155]. The large intestine or colonic inflammation induces diarrhea, which is referred to as colitis–complex diarrhea (CCD) in growing pigs, especially from 4–16 weeks post-weaning period. Pathogens such as *Brachyspira (B.) hyodysenteriae*, *B. pilosicoli*, and swine whipworms such as *Trichuris (T.) suis*, however, have been implicated in specific colitis (SC). Without specific pathogens, dietary factors such as high protein levels, pelleted feedstuffs, and a lack of antioxidants can cause non-specific colitis (NSC) [155].

Stress: Stressful situations such as overcrowding and poor sanitation can cause pigs to be more susceptible to infections, leading to disruption of the large intestine. The most common stressful condition pigs encounter during their life is related to transition during weaning (weaning-induced stress) which causes the dysbiosis of the gut microbiota [59,135]. A sudden change in a piglet’s diet or surroundings might disturb its intestinal microbial balance, putting them at risk for post-weaning diarrhea (PWD) [93,156].

### 4.3. Interventions or Prevention of Large Intestine Disruption across All Age Classes of Pigs

#### 4.3.1. Vitamins

The pig gut comprises approximately 1000 bacterial species, which are important to the host’s health [157]. These gut microbiotas are well known for their role in vitamin synthesis. Not only do gut microbiota synthesize B vitamins like biotin, vitamin B-12, folates, niacin, pantothenic acid, vitamin B-6, riboflavin, and thiamin, but they also synthesize vitamin K (Hill, 1997 as cited in [157]). Studies indicated that the *Bacteroidetes phylum* is the colon’s major bacterial community, accounting for more than 90% of vitamin B production (except for vitamin B-12) [157,158].

Dietary vitamin supplementation influences the composition and activities of the gut microbiota. According to Khan et al. [159], vitamin B2 can affect the growth of the anaerobic bacteria *Faecalibacterium prausnitzii*, which produces butyrate with anti-inflammatory properties. Anaerobic bacteria lack direct enzymatic defenses (e.g., catalase and peroxidase) against oxygen and reactive oxygen species (ROS). As a redox mediator, riboflavin can use metabolism to reduce the oxygenated environment, thereby reducing oxidative stress. In a study by Steinert et al. [160], volunteers received 100 mg of riboflavin daily for 14 days. They discovered that the amount of *Faecalibacterium prausnitzii* per gram of feces increased during supplementation and decreased once more, though not to baseline levels, after a 1-week washout. This study also discovered an increase in Roseburia species, another anaerobe, and a decrease in *E. coli* [146]. Recent research in pigs demonstrated that maternal 25OHD3 impacted the bacterial metabolites in the hindgut of the sucking piglets [161,162]. Sows supplemented with 25OHD_3_ altered the gut microbiota in the large intestine of suckling piglets [162].

#### 4.3.2. Amino Acids

Amino acids have been shown to significantly affect pig large intestine health by modifying the microbial profile, particularly the bacteria community. Dietary amino acids are important sources for microbiota fermentation in the pigs’ large intestines. Moreover, amino acids can be employed as a nitrogen source, enhancing the host’s development and the gut microbiota’s proliferation [163]. Furthermore, the pig’s gut’s microorganisms can catabolize amino acids to create metabolites such as kynurenine and indole that help the host’s feedback regulation [164]. An extensive review has been conducted on amino acids’ effect on pig gut health [5,163]. This section will focus on amino acids’ role in the pig’s large intestine health, which is linked with microbiota. Different amino acids have been studied on their effect on the gut microbiota, with threonine, lysine, methionine, tryptophan, and glutamine showing a positive effect by increasing the beneficial microbiota, reducing intestinal ammonia and opportunistic pathogens, as highlighted in the reviews by Ma et al. [163] and Liao [5].

Moreover, according to known mechanisms, amino acids affect gut microorganisms in several ways. The intake of amino acids encourages the production of intestinal b-defensin, endogenous cationic peptides, and other antibacterial compounds in the intestines, thereby limiting the growth of dangerous bacteria [164]. Apart from amino acids, the metabolites of amino acids as the results of microbiota metabolism are linked to large intestine health [153]. Strong intestine anti-inflammatory activity is exhibited by indole, the most prevalent microbial metabolite of aromatic amino acids, and its anti-inflammatory mechanism has been extensively researched [165,166]. A recent study on the effects of diets with different amino acid release properties on the gut microbiota of weaned pigs revealed that the diversity of the gut microbiota was increased by the balanced release of amino acid diets [167]. Furthermore, Zhao and his colleagues highlighted that dietary protein’s source, level, and amino acid balance of dietary protein primarily influence its composition, shape, and function. Over a diet with more protein than is necessary, an excellent protein-to-carbohydrate ratio or even a low-protein diet is advised. A rise in pathogenic microorganisms and an increase in the risk of metabolic disorders are caused by higher amounts of undigested protein [168]. This was further supported in a recent study where pigs were offered deficient protein diets supplemented with a mixture of Val above and Ile at NRC levels, which altered the gut microbiota by increasing the large intestine (colonic) Actinobacteria, Enterococcus, and Brevibacillus [169]. Amino acid supplementation affects the maternal and the offspring’s gut microbiota profile [170]. Metabolites produced from amino acids serve as critical molecular bridges between the gut flora and the host. Depending on their concentration, these metabolites from the gut microbiota might have advantageous or disadvantageous effects.

#### 4.3.3. Fibres/Prebiotics

The Prebiotic (pre-biosis) concept was introduced and defined by Gibson and Roberfroid as a “non-digestible food ingredient that beneficially affects the host by selectively stimulating the growth and/or activity of one or a limited number of bacteria in the colon and thus improves host health” [171]. Currently, a prebiotic is described as “a selectively fermented food that allows certain changes, both in the composition and (or) activity of microbiota, that bestow benefits upon host well-being and health” [172]. A feed component that significantly impacts this area is DF. Because endogenous digestive enzymes do not break down dietary fiber components, they are the primary substrates for bacterial fermentation in the large intestine [173]. Several reviews of dietary fiber’s role in gut microbiota have been intensively conducted [174,175,176]. These reviews highlighted that Western diets high in fat and sugar and poor in fiber cause beneficial Firmicutes that convert dietary plant-derived polysaccharides to SCFAs to decline and mucosa-associated Proteobacteria to grow (including enteric pathogens). Short-term diets can also have significant effects, especially those entirely animal-based, high-protein, and low-fermentable carbohydrate/fiber “weight-loss” diets [177]. These diets tend to increase the abundance of Bacteroides and decrease Firmicutes; long-term adherence to them will likely increase the risk of colonic disease. Fermentable prebiotic fibers that boost good *Bifidobacteria* or soluble fibers that limit bacterial-epithelial adhesion can be used to reduce intestinal inflammation (contrabiotics). These mechanisms could explain many variations in microbiota linked to a diet high in fiber from fruits and vegetables over the long term [176]. Changes in the fiber level and type of a pig’s diet significantly impact the structure of the bacterial community [178]. The large intestine’s bacterial community will change to accommodate the delivery of significant amounts of dietary fiber (as *Ruminococcus* spp., *Bacteroides* spp., and *Clostridium* spp. grow more often there) [174]. In another study, it was further noted that increased fiber intake appeared to facilitate hemicellulose digestion. Additionally, an increase in fiber consumption boosted the number of bacteria in the feces belonging to the families *Prevotellaceae*, *Ruminococcaceae*, and *Lachnospiraceae* and lowered the number of *Streptococcus* [179].

Moreover, increased fiber consumption facilitated fiber digestion, increased short-chain fatty acid (SCFA) synthesis, and improved microbial pyruvate and butanoate metabolism [177]. In addition, various metabolites, including SCFA (mainly butyrate, acetate, and propionate), lactate, succinate, ethanol, and gases, are produced due to the intestinal gut bacteria’s microbial fermentation of prebiotics in the colon. As a result, the colon’s acidic environment can alter the microbiota’s composition, which aids in reducing the growth of some potentially harmful bacteria such as *E. coli*, *Clostridium*, *Streptococcus faecalis*, and *Proteus* and promoting the growth of others such as bifidobacteria, lactobacilli, and Eubacterium [180]. The role of DF and prebiotics in preventing large intestine disruptions in pigs have been linked with the balance of gut microbiota. Studies on the weaning piglets reported that dietary supplementation of sugar beet pulp, inulin, lactulose, and wheat starch favored the bacterial communities facilitating fermentation. Fermentable carbohydrates may improve the diversity and stability of the bacteria found in the colon while also promoting the growth of *Lactobacillus sobrius*, a novel and helpful member of the commensal microbiota of pigs [181]. Prebiotics are believed to positively affect the body by selectively stimulating the growth and functions of bacteria important for a healthy gut, including *bifidobacteria*, *lactobacilli*, and *eubacteria* [149].

#### 4.3.4. Probiotics

Probiotics significantly enhance the pig’s gut health by regulating the gut microbiota homeostasis and maintaining the intestinal chemical and immunological barriers, as reviewed by [182]. According to Su et al. [182], the regulatory effects of probiotics on piglet postweaning diarrhea via the intestinal microbial barrier are primarily comprised of three aspects: (1) beneficial bacteria shape the gut microbiota; (2) pathogens are excluded through competitive exclusion; and (3) antimicrobial substances are produced and neutralize toxins. With the help of beneficial bacteria, the gut microbiota can be shaped by probiotics to become more diverse and resistant to harmful microorganisms [156]. A significant role of probiotic supplementation is maintaining healthy gut microbiota. Several studies have reported the relative abundance of *Lactobacillus* or *Bifidobacterium spp*. increased, *E. coli* decreased, and short-chain fatty acids (SCFAs) were produced at a higher rate in the gut of weaning piglets when lactic acid bacteria (*Lactobacillus johnsonii*, *Lactobacillus plantarum*, *Lactobacillus delbrueckii*, and *Enterococcus faecalis*) were added in their diet [182,183,184,185,186]. SCFAs, which colonic microorganisms generate by fermenting indigestible fiber, are crucial for immune system health, gut integrity, and glucose metabolism [187]. Several studies on the roles of probiotics have been conducted in pigs, not only in healthy pigs but also in treating or preventing diarrhea induced by antimicrobials [185], bacterial infection [155,181,183], and rectifying the large intestine dysbiosis caused by other stressors [182]. Adding probiotics to the maternal diets affects the maternal colonic microbiota and influences the offspring’s gut microbiota profile and metabolites [163,188]. Furthermore, maternal consumption of a fermented diet protected neonates from colonic inflammation by facilitating the maturation of the gut microbiota and proliferation of gut lactobacillus [189]. The strong correlations between the microbiota compositions of sows and their offspring are proof of maternal imprinting, which has benefits that persist after weaning [190].

#### 4.3.5. Other Dietary Factors

##### Resistant Starch

Several studies have been conducted on the influence of resistant starch (RS) on the composition and activity of microbes in the large intestine. According to the review by Tan et al. [191], a diet with increased resistant starch promotes the proliferation of beneficial bacteria and decreases the pathogenic bacteria in the large intestine, regardless of the age of the studied animal. Several studies demonstrate that dietary RS may improve gut health by increasing markers of mucosal barrier function, immune tolerance, and the diversity of beneficial intestinal microbiota [191].

##### Enzymes/Proteinase

Studies on the role of enzymes in modulating the gut microbiota indicate that feed enzymes may act on the undigested substrates, reducing the substrates for fermentation by gut microbiota. In addition, some enzymes may act directly on the outer membrane of the microbiota (e.g., dephosphorylation by alkaline phosphate) [192]. Based on numerous studies’ results, two mechanisms have been proposed for the influence of feed enzymes on the gut microbiota: reduction of an undigested substrate and production of short-chain oligosaccharides with potential prebiotic properties [192]. Furthermore, feed enzymes may also improve gut health by lowering intestinal viscosity due to soluble non-starch polysaccharides (NSP), which may slow digestion, increase digestive enzyme diffusion, and increase endogenous gut protein secretions. This, in turn, will increase substrate availability for microbial proliferation in the lower gut [134]. A recent study has demonstrated that dietary carbohydrases could alter the gut microbiota and significantly reduce the ammonia and fecal SCFAs and Indole content [193]. It was further noted that dietary enzymes such as carbohydrases could change not only the large intestine microbiota of the lactating sows but their piglets [194]. Based on numerous studies conducted either using multi-enzyme mixture [195,196] or single enzymes in the respective diets [193,194,197,198,199], regardless of the age class of a pig, they have been shown to improve the gut health by increasing the abundance of the large intestine microbiota associated with gut health and significantly improve the performance of weaned pigs [200].

Dietary lysozyme enzyme supplementation in pigs has been shown to facilitate the interaction between the gut microbiota and the host’s immune system. Increasing lysozyme supplementation in piglets increased the beneficial microbes in the colon, their relative abundance, and their metabolic functions [201]. However, one study observed that supplementing 100 mg/kg of lysozyme reduced the caecal microbiota abundance than in low doses or a control group in growing pigs [202]. The effect of dietary lysozyme supplementation in microbiota may be dose-dependent and influenced by the animal’s age and the method used to analyze the microbiota between the two studies. In addition, dietary lysozyme supplementation improves the beneficial microbe’s evenness and enhances gut immunity in weanling piglets [202,203]. Currently, there is an increasing body of information that dietary supplementation also enhances gut health under normal conditions, and lysozyme could prevent adverse effects against ETEC infection in weaned pigs [204] and neonatal piglets [205]. As reviewed by Oliver and Wells [206], the effect of lysozyme to decrease the severity of ETEC infection seems variable and may be influenced by the sources of the enzymes used in the studies in challenged piglets.

##### Plant Extracts

Furthermore, numerous studies have been conducted on the effects of plant extracts such as herbal extracts [207,208], essential oils, and organic acids on gut microbiota and on preventing diarrhea [148,209,210]. These feed additives are being recognized because they decrease the prevalence of pathogenic microbes in the cecum of weaned piglets. Dietary benzoic acids have postulated the same by affecting the cecal microbiota diversity and reverting post-weaning diarrhea in piglets [198,211,212,213]. A recent study was performed on an antimicrobial blended feed additive with essential oils, medium-chain fatty acid, and toxin-adsorbing minerals on weaned pigs challenged with ETEC [210]. The antimicrobial blend significantly decreased the occurrence of severe diarrhea in the weaned piglets and increased the relative abundance of beneficial bacteria while discouraging the pathogenic bacteria in the cecum [210,214]. Different dietary combinations of organic acids and fatty acids [215], plant extract and benzoic acid [216], or herbal mixtures [207,208] have been further tested and showed a positive effect in changing the large intestine microbiota, which could finally treat diarrhea in the weaned piglets.

Moreover, proanthocyanidin extract from plants has been showing a significant role in improving gut health by affecting lipid metabolism by increasing microbial propionate production in weaned pigs [217]. In addition, several studies have investigated the anthelmintic capabilities of proanthocyanidins and reported that in vitro tests using proanthocyanidin extracts from different plant sources showed it to be highly effective against the worm *A. suum* [218,219]. A current review by Andersen-Civil and his colleagues [220] highlighted how dietary proanthocyanidins could change the immunity towards harmful enteric microbes and parasitic infection by acting as the mediating factor in modulating the gut microbiota.

## 5. Conclusions

In the stomach, ulceration seems to be the major threat in intensive pork production. The knowledge about the roles of gastric digestion is increasing, but more research is needed, especially in connection to the effect on lower GIT parts. Due to the premature gut of piglets, they are highly vulnerable to small intestine disruptions caused by several stressors, such as weaning and pathogens. Adult pigs are also vulnerable to such disruptions due to environmental stressors. Dietary intervention via different feeding strategies and supplementation of vitamins, micro-minerals, amino acids, enzymes, pre- and probiotics, organic acids, and secondary plant metabolites can address the problems associated with intestinal disruptions. However, it is important to note that different concentrations of these feed supplements are reported in the literature. Therefore, these feed additives’ dose, efficacy, and safety must be carefully considered. The large intestine and microbiota significantly affect the host’s physiology and health. Several nutritional factors affect the large intestine’s health, mainly due to the modulation of the gut’s beneficial microbiota, which directly and indirectly (through microbial metabolites) maintain the large intestine’s health.

## Figures and Tables

**Table 1:** Some examples of the effect of peptide/amino acid supplementation on piglets’ small intestinal health.

Animal Weight and Age	Stressor	Adverse Effects	Treatment	Dose and Supplementation Duration	Positive Effects	References
6.64 kg, 21 days	Weaning	Compromised intestinal integrity and high incidence of diarrhea	Alanyl-glutamine (dipeptide)	0.45% in the diet for 21 days	Decreased diarrhea incidence, increased villus height, and villus height to crypt depth in the duodenum, jejunum, and ileum. The activity of maltase is increased, and a tendency to increase sucrase activity in the jejuna mucosa.	[111]
8.67 kg, 28 days	Oxidative stress	Compromised intestinal morphology and function	Arginine	1.6% in the diet for 7 days	Increased villus height in the ileum and suppressed the inflammatory cytokine expression in the jejunum.	[112]
7.23 kg, 21 days	Weaning	Low intestinal barrier integrity and morphology	Threonine	0.14% in the diet for 14 days	Increased mRNA expression of tight junction proteins (Zonula occludens-1 (ZO-1) and claudin-1) and increased villus height and goblet cell density in villi and crypts in the jejunum. Improved the inflammatory status in the jejunum.	[113]

**Table 2:** Effects of different types of feed additives on the small intestinal health of piglets under specific stressors.

Animal Weight and/or Age	Stressor	Adverse Effects	Treatment	Dose and Duration	Positive Effects	Ref.
Enzymes						
7.76 kg	Weaning	Low barrier integrity	Xylanase	60 mg/kg in the diet for 28 days	Increased the mRNA expression of Zonula occludens-1 and B-cell lymphoma/leukemia- 2 (Bcl-2), increased Sig A(main immunoglobulin in mucus secretions) secretions in the jejunum. Indicating improved intestinal physical barrier and immune barrier function.	[136]
6.55 kg, 28 days	Weaning	Compromised intestinal morphology and barrier integrity.	Protease	300 mg/kg in the diet for 28 days	Improved intestinal morphology (high duodenal villus height). Improved digestive enzyme (trypsin and chymotrypsin) activities in the duodenum and jejunum. Improve intestinal barrier activity (increase in the mRNA expression of ZO-1 and claudin-1 in the duodenum and jejunum).	[137]
Prebiotics and Probiotics						
6.3 kg, 25 days	Orally administered ETEC K88 strain (2 × 10^9^ CFU mL^−1^)	Compromised intestinal morphology	Lactulose	10 g/kg in the diet for 18 days	Increased ileum villus height and a reduction of the pig major acute-phase protein (Pig-MAP) in serum.	[138]
6.99 kg, 28 days	Orally administered ETEC F18+ (2 × 10^9^ CFU/g)	post-weaning diarrhea	*L. acidophilus*, *L. casei*, *B. thermophilum* and *E. faecium*	Each strain: 0.25 × 10^8^ CFU/g, for 5 days	Reduced expression of TNF-a; increased jejunal villus height, and villus height-to-crypt depth ratio	[139]
7.09 kg	Orally administered with ETEC F4 (1 × 10^9^ CFU/ g)	Intestinal injury	*Clostridium butyricum*	(5 × 10^5^ CFU/g) for 15 days	Alleviated intestinal villi injury caused by ETEC F4 challenge	[140]
4.5 kg, 14 days	Early weaning	Dysbiosis and reduced antioxidant capacity	*Saccharomyces* *cerevisiae*	3.0 g kg–1 live yeast (4.3 × 10^9^ CFU/g), for 21 days	Reduced the numbers of *E. coli* in the ileum contents; increased serum SOD activity and jejunum mucosal Sig A secretions	[141]
28 days	Deoxynivalenol (DON) (4 mg/kg in the diet)	Enhanced intestinal permeability and villi damage	Bovine lactoferrin, plant defensins, and active yeast	0.4% CAP in the diet for 30 days	Improved intestinal morphology (high villus height/crypt depth in the jejunum and ileum and increased goblet cell number in the ileum). Promoted intestinal epithelial cell proliferation.	[142]
Organic acids						
8.63 kg	Weaning	Post-weaning diarrhea, oxidative stress, and low intestinal morphology	Formic, acetic, and propionic acid combined with medium-chain fatty acids	3 g/kg in the diet for 28 days	Improve serum immune, antioxidant indices, and intestinal morphology (increased villus height to crypt depth ratio in the jejunum and ileum). Decreased incidence of diarrhea and *E. coli* counts in feces.	[143]
8.64 kg	Weaning	The abundance of *E. coli* and compromised intestinal morphology.	OA (Provenia): Benzoic acid (50%), calcium formate (3%), fumaric acid (1%), rest are coating material: palm oil and silicon dioxide	1.5 g/kg in the diet for 28 days	Increased villus height and improved apparent total tract digestibility of nutrients. Promote abundance of intestinal microbiota and an increasing trend in endogenous antioxidant enzymes.	[144]
Plant secondary metabolites and essential oils						
6.21 kg, 21 days	Weaning	Disturbed small intestinal histology	Alginate oligosaccharide	100 mg/kg in the diet for 14 days	Improved the structure with villus height and villus height to crypt depth ratio, increased the goblet cell counts in the duodenum and jejunum. Increased Sig A density in the jejunum and decreased the early- and late-stage apoptotic cell percentages and the total apoptotic cell percentage in the jejuna epithelium.	[145]
6.3 kg, 21 days	Orally administered ETEC F18+ (10^10^ CFU per 3 mL dose in PBS)	Compromised intestinal health	Capsicum oleoresin (CAP), garlic botanical (GAR), or turmeric oleoresin (TUR).	10 ppm, for 5 days	CAP and GAR increased the expression of genes related to the integrity of membranes in infected pigs, indicating enhanced gut mucosa health. All 3 plant extracts reduced the expression of genes associated with antigen presentation or other biological processes of immune responses, an indication of attenuating overstimulation of immune responses caused by *E. coli*.	[146]
8.68 kg, 28 days	Weaning	Compromised intestinal health with expressed oxidative stress and reduced digestive enzyme activity.	Natural capsicum extract (NCE)	80 mg/kg in the diet for 28 days	Increased activity of α-amylase, lipase, and protease activities in the jejunal mucosa and lipase activity in the ileal mucosa. Increased expression of endogenous antioxidant enzymes (superoxide dismutase and catalase), anti-inflammatory cytokine (IL-10), and subsequent decrease in malondialdehyde in serum.	[147]
6.74 kg, 21 days	Weaning	Oxidative stress compromised intestinal health and immune function.	EO: Next enhance 150 premixes plus, Novus International, Inc.) is a coated product containing 2.5% thymol, 2.5% carvacrol, and 95% of the inert carrier. Protease (Cibenza DP100, Novus International, Inc.	300 mg/kg essential oil and 500 mg/kg protease in the diet for 14 days	EO decreased the serum concentration of tumor necrosis factor-alpha, while protease reduced the serum concentration of malondialdehyde. Protease also increased the villus height and the ratio of villus height to crypt depth in the duodenum and increased sucrose activity in jejuna mucosa. The synergistic effect of EO and protease was expressed in reducing inflammatory parameters in weaning-challenged piglets.	[148]

ETEC—Enterotoxigenic *E. Coli*; CFU—colony-forming unit.

## Data Availability

Not applicable.
